# Age and MEN2 outcome

**DOI:** 10.18632/aging.102030

**Published:** 2019-06-12

**Authors:** Frederic Castinetti

**Affiliations:** 1 Aix-Marseille Université, Institut National de la Santé et de la Recherche Médicale, Marseille Medical Genetics, Marseille, France; 2 Assistance Publique-Hôpitaux de Marseille (AP-HM), Department of Endocrinology, Marseille, France

**Keywords:** medullary thyroid carcinoma, pheochromocytoma, RET, multiple endocrine neoplasia type 2

Multiple endocrine neoplasia type 2 (MEN2) is a rare genetic tumor syndrome due to mutations of the proto-oncogene RET [1]. Medullary thyroid cancer (MTC) is the most common feature of MEN2. MEN2 is divided into 2 different entities: MEN2A and MEN2B, the latter including the most aggressive form of MTC: with the earliest age of lymph node metastasis and systemic metastases for MEN2B reported as 1 and 6 years, respectively, due to the presence of the p.M918T RET mutation (which accounts for 95% of all cases of MEN2B) [2]. MEN2A includes high and moderate risks of MTC aggressiveness, leading to a more or less delayed appearance of lymph node and systemic metastases in comparison with MEN2B. To avoid the risk of metastases, the American Thyroid Association has recommended different ages for performing thyroidectomy, based on the RET mutation [3]. For example, patients with MEN2B should be operated before the age of 1 year, while patients with high risk MEN2A should be operated before the age of 5 years. In MEN2, the age at diagnosis of MTC appears to be crucial in defining the chance of remission.

In addition to the age at diagnosis, the mutation of RET classically defines the aggressiveness of MTC. For instance, high risk MEN2A-MTC is suggested to be intrinsically more aggressive than moderate risk MEN2A-MTC. However, this hypothesis has recently been challenged: when taking into account the follow-up from the age at diagnosis and not the overall age at last follow-up, both survival and the risk of distant metastases were not significantly different between high and moderate risk MEN2A MTC. Age at diagnosis was thus considered as the most important marker of MTC outcome in MEN2A, emphasizing the need to perform early thyroidectomies [4]. This point had not previously been evaluated in a large cohort of MEN2B patients. Our large international multicentric study based on 345 patients confirmed that MTC was indeed diagnosed at an earlier age in MEN2B than in MEN2A: however, overall survival was much lower in MEN2B than in MEN2A, when comparing MEN2B and MEN2A patients from their age at diagnosis, suggesting that the p.M918T mutation was *per se* a strong marker of MTC aggressiveness. This was not surprising since the somatic p.M918T mutation was also found in aggressive forms of non-hereditary MTC, suggesting that this mutation *per se* was able to modify the aggressiveness of MTC. Interestingly however, 46 patients were cured by a single surgery, despite the fact that they had thyroidectomy far later than 1 year of age [5]. While age at diagnosis is thus a strong marker of MTC outcome, other factors such as RET polymorphisms have yet to be determined and may enable better understanding of this phenotypic variability.

The second main feature of MEN2 is pheochromocytoma, in almost all cases a benign tumor of the adrenal medulla, leading to a hypersecretion of metanephrines and normetanephrines. The penetrance of pheochromocytoma as well as the age at diagnosis is also dependent on the RET mutation. For example, we reported that half of the patients with a MEN2A 634 codon mutation (observed in roughly 80% of MEN2A cases) would present a single pheochromocytoma by age 32, while half of the patients with p.M918T MEN2B would present bilateral pheochromocytoma by the same age [5,6]. Biochemical screening methods are recommended at yearly intervals to detect the pheochromocytoma as early as possible. This is of major importance so that adrenal sparing surgery can be performed before the pheochromocytoma becomes too large [3]. This specialized surgery allows removal of the pheochromocytoma while maintaining a small amount of residual adrenal tissue to permit normal glucocorticoid function and avoid the risk of adrenal insufficiency.

It should be noted that MTC and pheochromocytoma are not the only factors impacting the life of patients with MEN2B. Extra-endocrine features, such as a marfanoid habitus, severe constipation, eye anomalies, or motor and muscle deficiencies can be detected at an early age in more than 50% of the patients with MEN2B, far earlier than the age at which endocrine signs will be apparent. These features partly explain why 25% of our MEN2B patients report, at last follow-up, being unable to work or attend school due to disability [5].

To conclude, the management of MEN2 requires a high level of expertise in order to propose the best therapeutic option at a given time point. Age-related guidelines should be followed whenever possible, but one should never forget that other, as yet unknown, factors may modify the natural history of the disease defined by the mutation of RET. In any case, the age at diagnosis is crucial for remission: this implies that all physicians should be aware of the extra-endocrine early signs of MEN2B to facilitate the earliest possible management of this aggressive syndrome.
